# The multifunctional role of SPANX-A/D protein subfamily in the promotion of pro-tumoural processes in human melanoma

**DOI:** 10.1038/s41598-021-83169-1

**Published:** 2021-02-11

**Authors:** Itziar Urizar-Arenaza, Aitor Benedicto, Arantza Perez-Valle, Nerea Osinalde, Vyacheslav Akimov, Iraia Muñoa-Hoyos, Jose Antonio Rodriguez, Aintzane Asumendi, Maria Dolores Boyano, Blagoy Blagoev, Irina Kratchmarova, Nerea Subiran

**Affiliations:** 1 Department of Physiology, University of the Basque Country (UPV/EHU), 48940 Leioa, Spain; 2 Biocruces Bizkaia Health Research Institute, Bizkaia, Spain; 3 Department of Cell Biology and Histology, University of the Basque Country (UPV/EHU), Leioa, Spain; 4 Department of Biochemistry and Molecular Biology, University of the Basque Country (UPV/EHU), Vitoria-Gasteiz, Spain; 5 Department of Biochemistry and Molecular Biology, University of Southern Denmark, Odense, Denmark; 6 Department of Genetics, Physical Anthropology and Animal Physiology, University of the Basque Country (UPV/EHU), Leioa, Spain

**Keywords:** Cancer, Cell biology, Molecular biology, Molecular medicine, Oncology

## Abstract

Human sperm protein associated with the nucleus on the X chromosome (SPANX) genes encode a protein family (SPANX-A, -B, -C and -D), whose expression is limited to the testis and spermatozoa in normal tissues and various tumour cells. SPANX-A/D proteins have been detected in metastatic melanoma cells, but their contribution to cancer development and the underlying molecular mechanisms of skin tumourigenesis remain unknown. Combining functional and proteomic approaches, the present work describes the presence of SPANX-A/D in primary and metastatic human melanoma cells and how it promotes pro-tumoural processes such as cell proliferation, motility and migration. We provide insights into the molecular features of skin tumourigenesis, describing for the first time a multifunctional role of the SPANX-A/D protein family in nuclear function, energy metabolism and cell survival, considered key hallmarks of cancer. A better comprehension of the SPANX-A/D protein subfamily and its molecular mechanisms will help to describe new aspects of tumour cell biology and develop new therapeutic targets and tumour-directed pharmacological drugs for skin tumours.

## Introduction

The SPANX family (sperm protein associated with the nucleus on the X chromosome) is a multigene family mapped to the X chromosome. SPANX genes encode proteins that belong to the so-called “cancer testis antigen” (CTA) family, a group of proteins whose expression is limited to the testis and spermatozoa in normal tissues and various tumours in nongametic cells^1,2^. SPANX proteins, similar to other CTAs, are exclusively expressed in post-meiotic haploid cells localised on the immune-privileged adluminal side of the haematotesticular barrier^1,3,4^. Because of their immunological characteristics, CTAs are considered the most promising candidates for cancer immunotherapy.

The SPANX gene family encodes two subfamilies in humans: SPANX-N and SPANX-A/D. The SPANX-N subfamily comprises SPANX-N1, N-2, N-3, N-4 and N-5^1^, and the SPANX-A/D subfamily comprises the SPANX-A1, -A2, -B, -C and –D isoforms^2,3,5^. All SPANX proteins exhibit a similar postmeiotical expression pattern during spermatogenesis and in mature spermatozoa^1,6,7^. Recently, we discovered that SPANX-A/D proteins are involved in numerous functions, including nuclear envelope organisation, sperm movement and metabolism^7^, suggesting their potential as promising targets for sperm fertility management.

In addition to their physiological function in sperm fertility, SPANX, similar to other CTAs, plays a pathological role because it is expressed in various tumours originating from nongametic cells^3,6^. Unlike other CTAs, SPANX-N subfamily is expressed not only in testis, but also in other non-tumour cells such as placenta, lung or colon, and in several cancers, such as melanoma, bladder carcinoma or myeloma^1^. However, SPANX-A/D proteins are normally present in the testis and overexpressed in several cancers, including haematological malignancies, myeloma, breast, bladder and prostate carcinomas, and melanoma^8–10^. Increased SPANX-A/D expression correlates with liver metastasis in colorectal cancer patients^11^ and has been detected in metastatic melanoma^9^, suggesting a potential role in the invasion and/or metastasis capacity of some tumours^12^. Remarkably, higher levels of CTA are associated with more aggressive skin tumours, such as non-lymphatic metastatic melanomas^9^. Nevertheless, the molecular mechanisms driving the malignant differentiation of tumour cells remain unknown.

Because growing evidence points to SPANX-A/D proteins as possible mediators of cancer development, we aimed to elucidate the pathological role of the SPANX-A/D protein subfamily in skin tumourigenesis. Combining proteomics, molecular and cell biology approaches, we describe for the first time the multifunctional role of the SPANX-A/D protein family in human melanoma. By scaffolding specific proteins, SPANX-A/D proteins may regulate several hallmarks of cancer, including nuclear functions and organisation, energy metabolism and cell survival, to promote pro-tumoural processes such as proliferation, motility and migration.

## Results

### The SPANX-A/D protein subfamily is expressed in human melanoma cells

**Figure 1:**
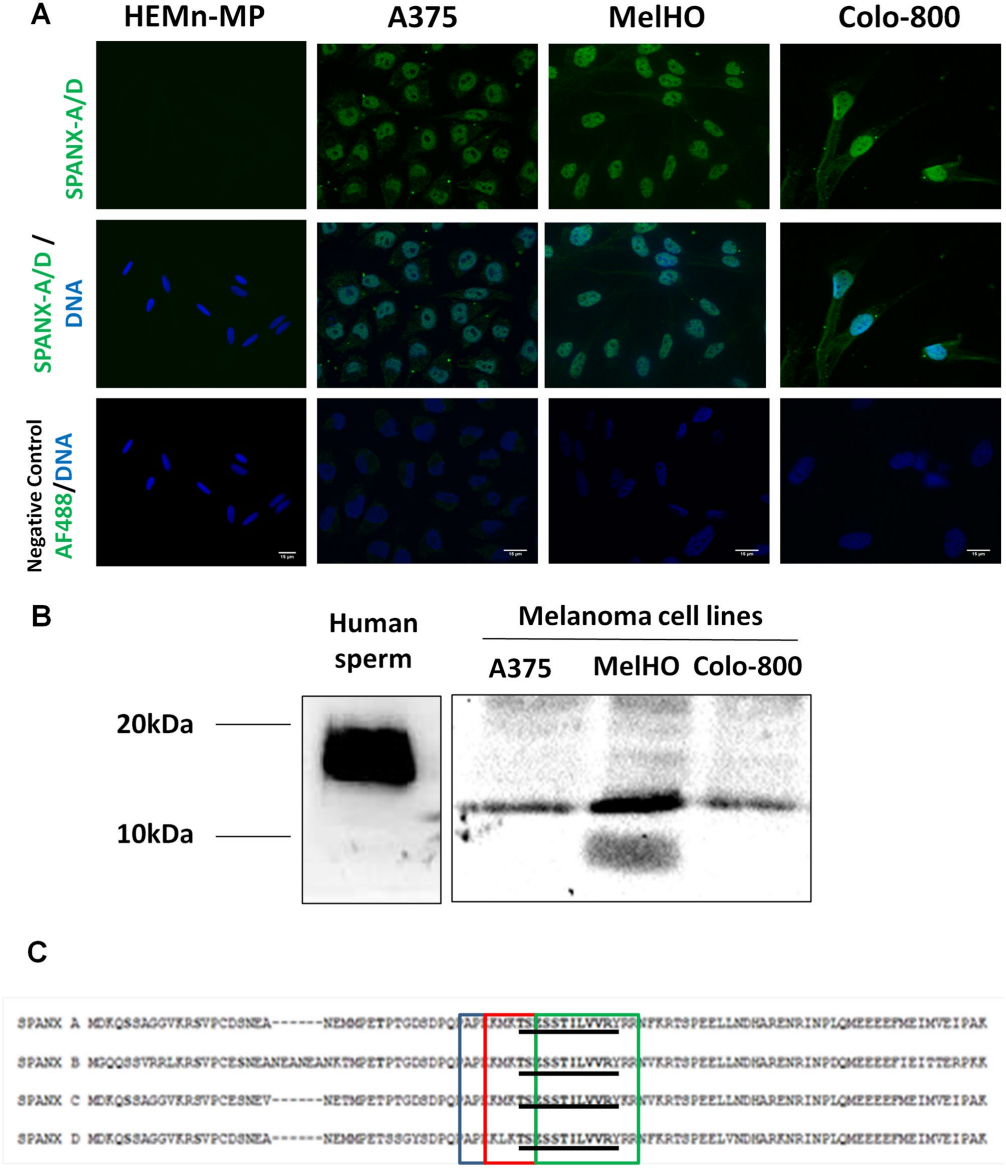
Characterisation of SPANX-A/D protein family in human melanoma cancer cells. **(A)** by immunocytochemistry. A375, MelHO and Colo-800 human melanoma cancer cell lines were used. As negative control cells HEMn-MP melanocytes were used. For the specificity of the secondary antiserum, the primary antibody was omitted. The nuclei were stained with Hoechst and are represented in blue. Scale bar 15 μm. AF488: Alexa Fluor 488 donkey anti-rabbit IgG (Thermo Scientific) (N = 4). Images were evaluated using ImageJ software (1.48v) **(B)** by western blotting technique. A375, MelHO and Colo-800 human melanoma cancer cell lines were used. Human sperm lysate was used as positive control. (N = 3). Crop from Supplementary Fig. S2. **(C)** Comparison of each SPANX isoform sequences with the three overlapping consensus nuclear localisation signals remarked in shaded boxes. The peptide detected by LC–MS/MS is underlined for all isoforms.

SPANX-A/D proteins belong to the so-called CTAs that are physiologically expressed in normal male germ cells and aberrantly expressed in various cancers^6,13^. Immunofluorescence analyses were performed to evaluate the presence of the SPANX-A/D protein subfamily in several cancer cell lines. The SPANX-A/D subfamily was highly expressed in A375, MelHO and Colo-800 melanoma cell lines (Fig. 1A), as well as in several cell lines derived from distinct tumours, such as SW480 and HCT-8 (colorectal adenocarcinoma), HeLa (epithelioid cervix carcinoma), A2780 (ovary adenocarcinoma), SMS-KCNR (neuroblastoma cell line), and MCF-7 (mammary adenocarcinoma) (Supplementary Fig. S1). Remarkably, in all the tested cell lines, SPANX-A/D immunolabelling was prominently nuclear, with a faint cytoplasmic signal noted in some cases (Fig. 1A). As we expected, no signal was observed in human melanocytes (HEMn-MP), proving the specificity of the primary antibody. In negative control samples, in which incubation with the primary antibody was omitted, fluorescence was not observed.

A more in-depth analysis of the melanoma cell lines by immunoblotting confirmed the presence of SPANX-A/D proteins in both primary (A375 and MelHO) and metastatic (Colo-800) melanoma cell lines (Fig. 1B). The anti-SPANX polyclonal antibody labelled an 11-kDa band. No immunoreactivity was observed when the primary antibody was omitted (data not shown). To further characterise the expression of the SPANX-A/D subfamily in melanoma cells, we sought to determine the presence of different protein isoforms in A375 cells. We detected a single peptide (TSESSTILVVR) that corresponds to a nuclear localisation signal (NLS) shared by all SPANX-A, -B, -C and -D isoforms (Fig. 1C). Hence, proteomic analysis corroborated the presence of SPANX-A/D in A375 cells but could not determine whether one, two, three or all members were co-expressed. According to our results, SPANX-A/D can be phosphorylated in human spermatozoa^7^. To uncover whether this subfamily is also phosphorylated in A375, we conducted proteomic studies. Notably, no phosphorylated SPANX-A/D residues were found in this analysis, suggesting that these proteins are mostly in a non-phosphorylated form in A375 cells.

### SPANX-A/D expression promotes the proliferation, motility and migration of human melanoma cells

**Figure 2:**
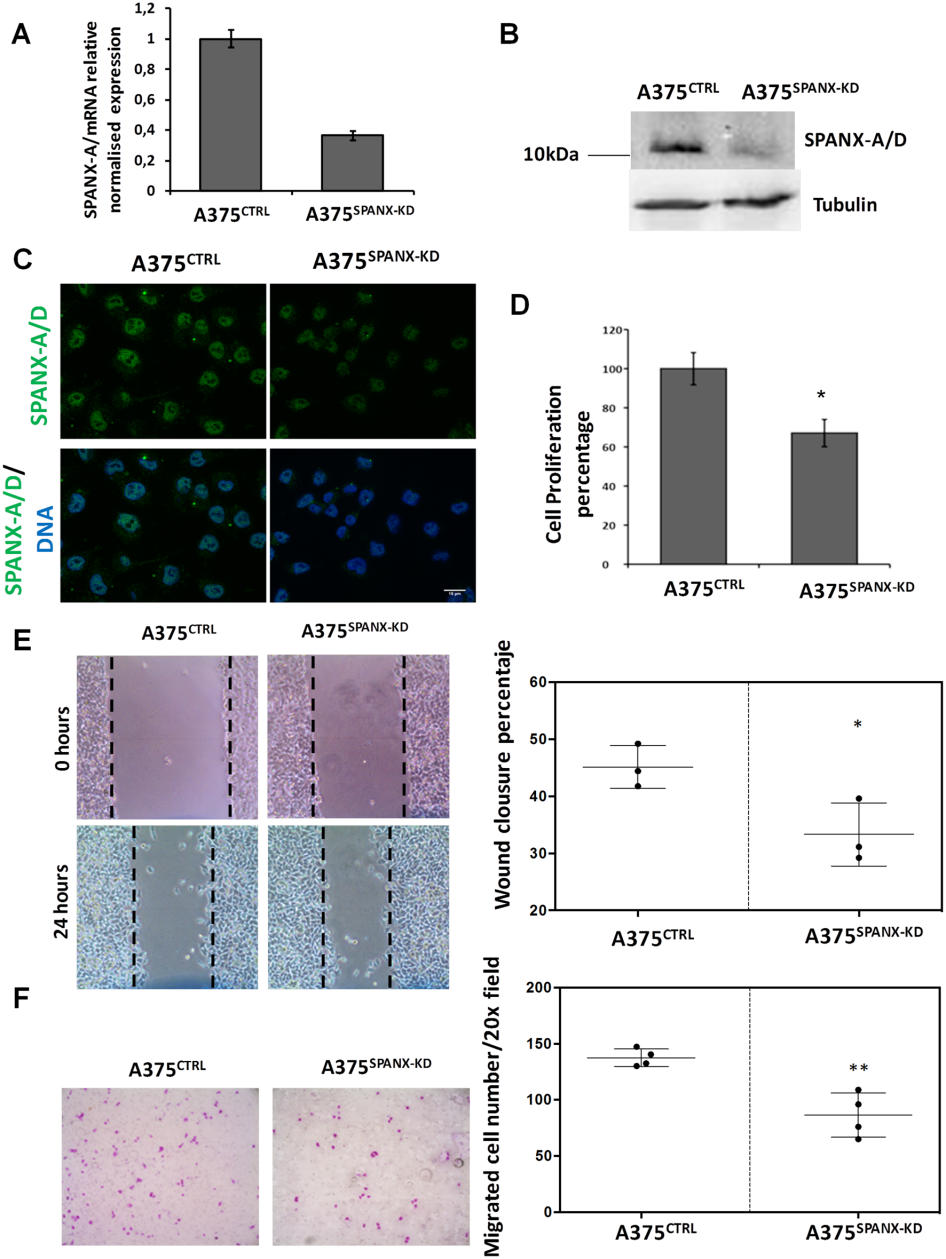
Role SPANX- A/D protein family in proliferation, motility and migration of A375 human melanoma cell line. Silencing of SPANX-A/D **(A)** at mRNA level by RT-qPCR assays **(B)** at protein level by western blotting (Crop from Supplementary Fig. S3) and **(C)** immunocitochemistry assays after transfecting A375 cells with shRNA targeting SPANX-A/D (A375^SPANX-KD^). A375^CTRL^ represent cells transfected with scramble shRNA. The nuclei were stained with Hoechst and are represented in blue. Scale bar 15 μm. (N = 3). Effect of the A375 cells transfected with shRNA SPANX-A/D in **(D)** Cell proliferation. Results are presented as the percent proliferation comparing A375^CTRL^ vs A375^SPANX-KD^ cells. Images were evaluated using ImageJ software (1.48v) **(E)** Cell migration. Photographs were taken at T = 0 to use them as initial wound area and after 24 h of incubation (T = 24 h), using inverted light microscope. Results are presented in a graph as wound closure percentage comparing A375^CTRL^ vs A375^SPANX-KD^ cells. The closed area was calculated using the ImageJ software (1.48v) by means of initial wound area at T = 0–T = 24. **(F)** Cell Trans-well migration. The results are expressed as the average of total number of migrated cells in 20 × field and compared A375^CTRL^ vs A375^SPANX-KD^ cells. The number of invading cells was quantified by counting stained cells in random fields of the membrane. The data shown in graphs **(D)**, **(E)** and **(F)** correspond to the mean of three independent experiments, and error bars indicate the SEM. *P < 0.05; **P < 0.01 (Students T-test).

Recently, SPANX-A/D proteins have emerged as strong candidates for cancer immunotherapy^14,15^. However, the role of this family in skin tumourigenesis remains largely unknown. SPANX-A/D proteins have been detected in metastatic melanoma cells^9^, but their contribution to metastatic development and the potential molecular underlying mechanisms have not yet been determined. To further investigate the potential significance of SPANX-A/D in the proliferation and migration capacity of melanoma cells, we used lentiviral-mediated delivery of short hairpin (sh) RNAs to generate stable knockdown variants of the A375 human melanoma cell line with reduced expression of SPANX-A/D (A375^SPANX-KD^) and a control cell line (A375^CTRL^). We generated and checked three different shRNAs, and the most effective shRNA was used for further experiments (data not shown). The depletion of SPANX-A/D was confirmed at the mRNA and protein levels by qRT-PCR and immunoblotting, respectively (Fig. 2A,B). A reduction of approximately 65% in SPANX-A/D mRNA and protein levels was observed in cells transfected with the most effective shRNA compared with scramble shRNA-transfected cells. Furthermore, the SPANX-A/D immunofluorescence signal was absent from the nucleus of A375^SPANX-KD^ cells (Fig. 2C).

We first analysed tumour cell proliferation using the PrestoBlue viability assay. Reduced expression of SPANX-A/D resulted in the decreased cell viability of A375 cells. A375^SPANX-KD^ cells exhibited 35% reduced proliferation following 24 h of incubation compared with A375^CTRL^ cells (Fig. 2D). Additionally, we analysed the ability of SPANX-A/D to modulate A375 tumour cell motility. Wound healing assays revealed reduced motility of SPANX-A/D knockdown cells (Fig. 2E). A375^CTRL^ cells closed 45% of the wound, while A375^SPANX-KD^ cells only closed 35% of the scratch. Therefore, SPANX-A/D deficiency led to a 10% reduction in wound closure. Finally, we conducted Transwell assays to evaluate the Transwell migration capacity of SPANX-A/D knockdown cells. An average of 140 A375^CTRL^ cells per 20 × field migrated through an 8-µm pore filter, whereas an average of only 90 A375^SPANX-KD^ migrated through the filter (Fig. 2F). Therefore, according to our results, the Transwell migration capacity of SPANX-A/D-deficient melanoma cells was reduced by approximately 35% relative to that of control cells. Taken together, our results show that the depletion of SPANX-A/D compromises the capacity of A375 melanoma cells to proliferate and migrate.

### SPANX-A interacts with proteins involved in crucial nuclear processes in melanoma cells

**Figure 3:**
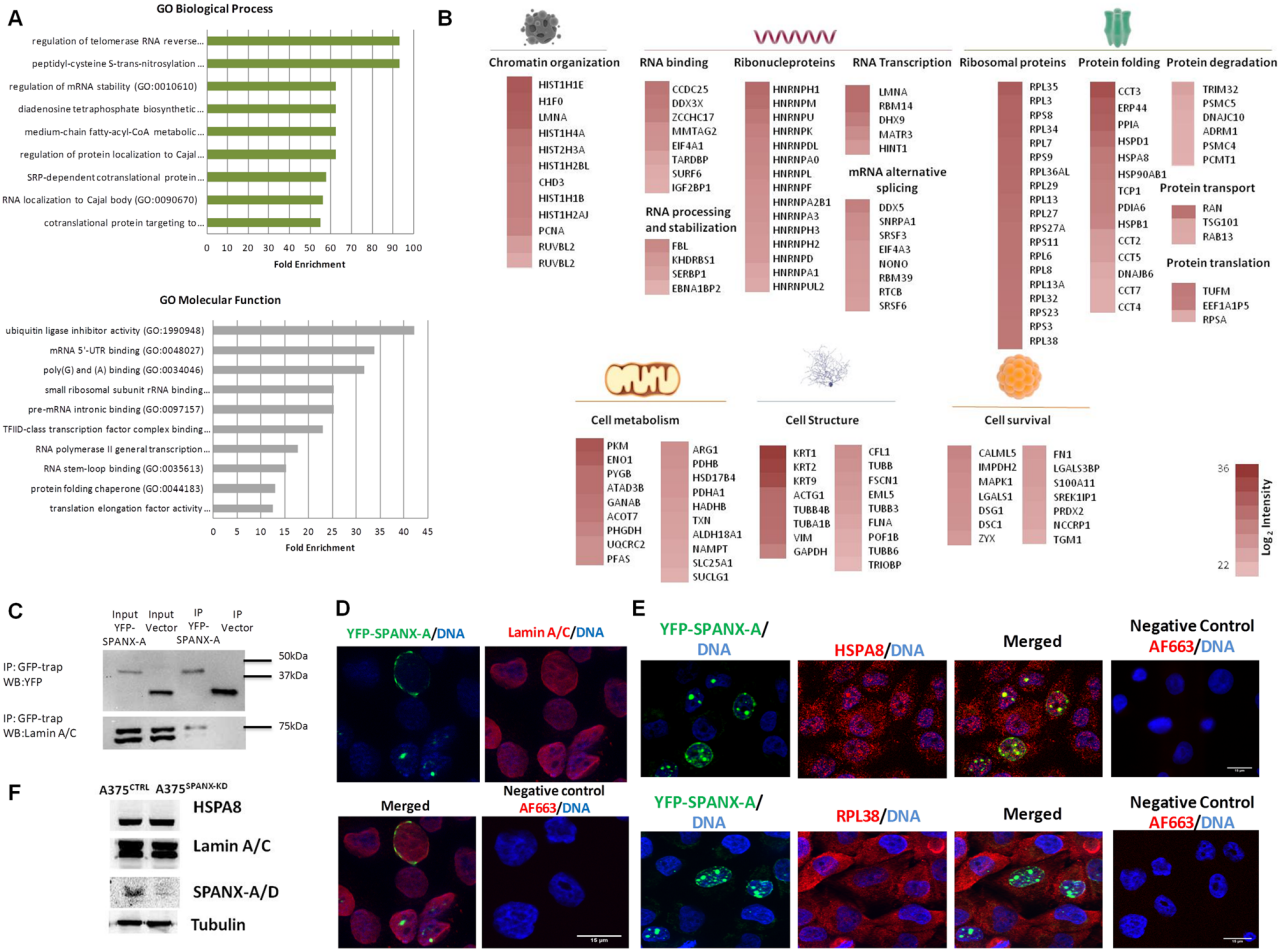
Study of the YFP-SPANX-A interactome A375 human melanoma cell line. **(A)** Gene Ontology based on the biological function and molecular processes of co-precipitated proteins together with SPANX-A in A375 melanoma cell line. The ten most enriched processes are shown. P < 0.05 **(B)** Representative scheme of principal putative interactors of SPANX-A based on their function. Information was obtained from Uniprot Database. Only the most representative functions are shown. Only the 19 most intense ribosomal proteins are shown **(C)** Validation of the interaction between YFP-SPANX-A and Lamin A/C by co-immunoprecipitation and western Blotting (N = 3). Crop from Supplementary Fig. S4. **(D)** Validation of the co-localisation between YFP-SPANX-A and Lamin A/C by immunofluorescence. YFP-SPANX-A is represented in green and Lamin A/C is shown in red. For the specificity of the secondary antiserum, the primary antibody was omitted. The nuclei were stained with Hoechst and are represented in blue. Scale bar 15 μm. (N = 3) Images were evaluated using ImageJ software (1.48v) **(E)** Co-localisation between YFP-SPANX-A and HSPA8, and YFP-SPANX-A and RPL38 by immunofluorescence**.** For the specificity of the secondary antiserum, the primary antibody was omitted. The nuclei were stained with Hoechst and are represented in blue. Scale bar 15 μm. (N = 3) Images were evaluated using ImageJ software (1.48v) (**F**) Expression of Lamin A/C and HSPA8 in A375^SPANX-KD^ cells. A375^SPANX-KD^: Stable SPANX-A/D knockdown variant of the A375 human melanoma cell line; A375^CTRL^: control cell line. N = 3.

To gain insight into the molecular mechanisms by which SPANX-A/D might promote proliferation and migration in melanoma cells, we aimed to study the interactome of SPANX-A. Briefly, YFP-SPANX-A was expressed in A375 cells and pulled down in quadruplicate using GFP-Trap. All the precipitated proteins were analysed by mass spectrometry and subjected to label-free quantitative proteomics. We detected 246 potential interactors of SPANX-A (Supplementary Table S1). To investigate the biological and molecular functions in which these putative SPANX-A interactors might be involved, we performed two different gene ontology analyses using the PANTHER functional annotation tool. SPANX-A seems to participate in various biological processes, including the regulation of mRNA processing and stabilisation, cell metabolism and the transport of proteins and RNA to Cajal bodies and other cell compartments (Fig. 3A). Regarding molecular function, the most enriched processes were those related to RNA transcription, mRNA binding and alternative splicing, and protein translation and folding. Taken together, these results highlight the possible involvement of SPANX-A in crucial nuclear processes.

To obtain more detailed idea about the nature and roles in which putative SPANX-A proteins are involved, we checked them in the UniProt database (Supplementary Table S2). Based on the data collected in the curated database, all the proteins that constitute our list of putative SPANX-A binding proteins were classified into 14 categories (Fig. 3B). Consistent with the results obtained in the gene ontology analysis, most of the proteins are involved in key nuclear functions, such as chromatin organisation, RNA transcription and protein translation. Notably, our list of putative SPANX-A proteins includes 15 ribonucleoproteins and 64 ribosomal proteins. Additionally, of the 246 putative SPANX-A interactors, 19, 17 and 14 proteins were involved in cell metabolism, cell structure and cell survival, respectively.

Next, we validated the SPANX-A interaction with proteins related to nuclear functions, such as Lamin-A/C (LMNA), one of the proteins that showed the highest MS intensity. Co-immunoprecipitation analyses confirmed the physical interaction between both proteins. LMNA was detected in YFP-SPANX-A-containing immune complexes (Fig. 3C) and co-localised with SPANX-A over the nuclear envelope (Fig. 3D). Consistent with the results obtained in gene ontology analysis, we also identified a broad collection of proteins related to protein translation and folding, such as ribosomal proteins and chaperones (Fig. 3B). Specifically, immunofluorescence analyses confirmed the co-localisation of YFP-SPANX-A and 60S ribosomal protein L8 (RPL38) in the cytoplasm and heat shock cognate 71 kDa protein (HSPA8) in the nucleus of A375 cells (Fig. 3E).

Finally, among the 246 putative SPANX-A interactors, we found several proteins involved in the following processes: cell metabolism, such as pyruvate kinase (PKM), D-3-phosphoglycerate dehydrogenase (PHGDH) or pyruvate dehydrogenase E1 component subunit alpha (PDHA1); specific enzymes that participate in glucose catabolism; cell structure proteins related to the cytoskeleton, such as actin (ACTG1), α/β tubulins (TUBA1B and TUBB4B) or keratins (KRT1, KRT2 AND KRT9); and cell survival, such as mitogen-activated protein kinase 1 (MAPK1), inosine-5-monophosphate dehydrogenase 2 (IMPDH2) or F-box only protein 50 (NCCRP1).

To provide further information about the molecular mechanisms of SPANX-A/D in the promotion of tumorigenic processes, we evaluated the expression of Lamin A/C and HSPA8 in SPANX knockdowned melanoma cells (A375^SPANX-KD^). As can be observed in Fig. 3F, the expression of both Lamin A/C and HSPA8 did not change in A375^SPANX-KD^ cells, indicating that the expression of both interactors is not dependent to SPANX-A/D expression. Our results, therefore, indicate that the interaction between SPANX-A/D and these proteins is crucial for the promotion of tumourigenic processes in human melanoma, rather than the regulation of their protein expression.

## Discussion

**Figure 4:**
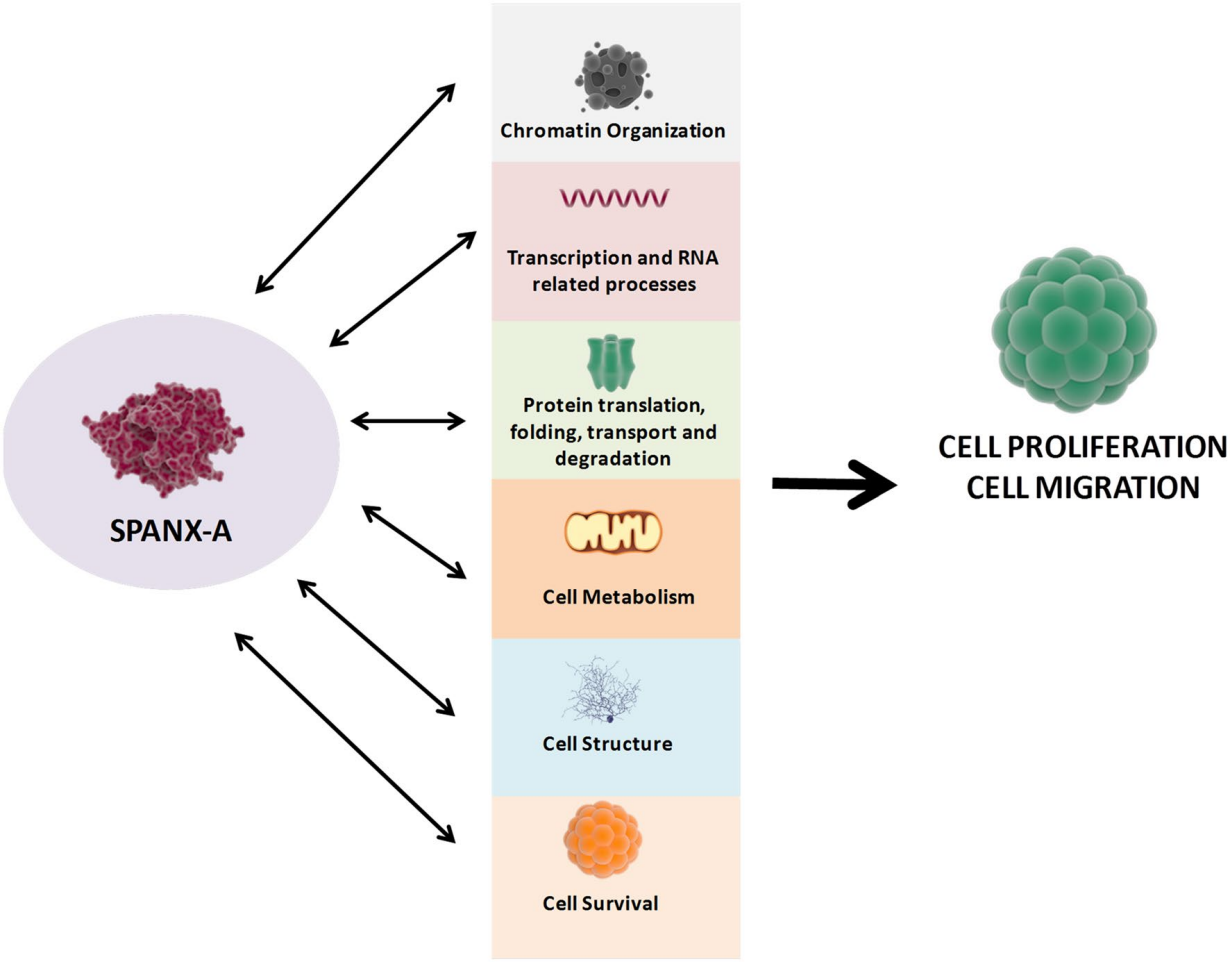
Role of SPANX-A in human melanoma. Representative scheme of the multifunctional role of SPANX-A in tumor progression. SPANX-A it is mainly involved in multifunctional processes like chromatin organisation, transcription and RNA related processes, protein translation initiation, protein folding, transport and degradation, cell metabolism and structure, which promote cell proliferation, motility and migration of cancer in human melanoma.

Over the last twenty years, many studies have analysed the presence of the SPANX-A/D subfamily in various cancers, such as breast, myeloma, haematological, melanoma, bladder and prostate carcinomas^3,8–12^, to clarify the function of the protein subfamily in carcinogenesis. Through a combination of functional and proteomic approaches, we proved that SPANX-A/D proteins play a multifunctional role in skin tumourigenesis, providing insight into how this protein family may promote pro-tumoural processes in human primary melanoma cells (Fig. 4).

We confirmed the presence of SPANX-A/D proteins in different cancer types, such as colorectal cancer, cervical-uterine cancer, neuroblastoma, mammary cancer and melanoma cells. Although higher levels of SPANX-A/D are reported to be associated with more aggressive skin tumours^9^, our results show the presence of the SPANX-A/D protein family in both primary and metastatic melanoma cell lines presenting nuclear localisation. However, we have previously reported that the NLS of SPANX-A/D proteins is strongly phosphorylated in human spermatozoa^7^. However, surprisingly, we could not find any phosphorylated SPANX-A/D residues at the NLS in A375 cells. Considering that the SPANX-A/D protein family is mainly localised in the nucleus of melanoma cell lines, our findings confirm that the phosphorylation state of the NLS is not crucial for nuclear SPANX-A/D translocation either in spermatozoa^7^ or in A375 cell lines.

SPANX-A/D proteins play a role in the invasion and/or metastasis of several tumours. Our results, together with other studies, describe the presence of SPANX-A/D proteins in metastatic melanoma^9^, and their expression correlates with liver metastasis in colorectal cancer patients^11^. Given the potential significance of SPANX-A/-C/-D in the proliferation and migration^12^ of breast cancer cells, we further investigated the function of SPANX-A/D proteins in melanoma cells. The lack of the protein family in the A375 cell line led to reduced proliferation, cell motility and migration, indicating that the SPANX-A/D protein family promotes different pro-tumoural processes, as previously described in breast and lung cancer^12,16^. Uncontrolled cell proliferation and migration represent the essence of neoplastic disease. To elucidate the underlying mechanisms of the pathological role of SPANX-A/D in these pro-tumoural processes, we performed interactome analysis of YFP-SPANX-A overexpression in the A375 melanoma cell line. SPANX-A co-immunoprecipitates with 246 proteins that are involved in certain hallmarks of cancer^17^, such as nuclear functions, metabolism and cell survival. SPANX-A co-precipitates with proteins involved in cell growth and proliferation (such as mitogen-activated protein kinase 1 (MAPK1) and F-box only protein 50 (NCCRP1)), cell adhesion (such as carcinoma-like desmoglein-1 (DSG1) and desmocollin-1 (DSC1), which are involved in different types of cancer^30–33^ and cytoskeletal proteins (such as actin, tubulins and keratins, which are associated with the capacity of cells to migrate and invade new tissues^18^).

However, the SPANX-A/D protein family may be involved in other uncharacterised biological functions in melanoma cells. According to our proteomic analysis, the SPANX-A/D protein family plays a multifunctional role in melanoma cells, as we have previously reported in human spermatozoa^7^. Considering that this protein family is mainly expressed in the nucleus of the A375 melanoma cell line, SPANX-A interactors that are involved in nuclear functions show the highest intensity values. SPANX-A seems to participate in various nuclear processes, including chromatin organisation, RNA processing and stabilisation, RNA binding, alternative splicing and RNA transcription. Our results show that SPANX-A interacts with LMNA, a finding that is consistent with previous studies performed with SPANX-C^12^, and both proteins co-localise at the nuclear envelope. LMNA is a component of a meshwork of nuclear lamina proteins that underlie the inner nuclear membrane and provide structural stability to the nucleus^19^. LMNA, which is a gene expression regulator, modulates chromatin accessibility and influences the ability of transcription factors to interact with DNA^20^ by scaffolding the formation of multiprotein complexes^21^. SPANX-A/D may induce cell proliferation and migration in melanoma cells by forming multiprotein complexes that alter nuclear function because SPANX-A not only interacts with LMNA but also coimmunoprecipitates with several chromatin regulators, such as histones, and a wide range of RNA processing proteins involved in RNA stabilisation, translation and alternative splicing, among others. Because aberrant gene expression and abnormal functioning of nuclear processes have been extensively associated with cancer progression in different tumours^22–24^, our findings indicate that SPANX-A/D may play a role as a scaffold of multi-protein complexes and recruit proteins related to several nuclear functions to promote pro-tumoural processes in melanoma cells.

However, we also identified a broad collection of proteins involved in protein translation, folding and targeting to cell compartments, such as ribosomal proteins or chaperones, as potential SPANX interactors. Specifically, the HSPA8 chaperone and ribosomal protein RPL38 co-localise with SPANX-A in the nucleus and cytoplasm of the A375 cell line, respectively. HSPA8 is usually expressed in the cell nucleoplasm and has been proposed as a biomarker for the early diagnosis of endometrial carcinoma^25^. This chaperone belongs to the spliceosome complex^26^, which is involved in mRNA splicing^27,28^, providing new insights into the role of SPANX A/D in mRNA alternative splicing. Although correct protein folding is essential for the proper functioning of cells, protein transport is not less important. Protein transport regulators are crucial in the mediation of cancer cell biology, encompassing uncontrolled cell growth, invasion and metastasis^29^.

Additionally, SPANX-A also co-precipitates with proteins involved in cell metabolism. The capability to modify or reprogram cellular metabolism to most effectively support neoplastic proliferation is an emerging hallmark of cancer^17^. Otto Warburg described for the first time the anomalous functioning of energy metabolism in cancer cells^30,31^. Cancer cells can reprogram their glucose metabolism, and thus their energy production, by limiting their energy metabolism largely to glycolysis rather than mitochondrial oxidative phosphorylation, leading to a lower efficiency of ATP production even in the presence of oxygen. SPANX-A co-precipitates with specific enzymes that participate in glucose catabolism, such as pyruvate kinase (PKM), D-3-phosphoglycerate dehydrogenase (PHGDH) and pyruvate dehydrogenase E1 component subunit alpha (PDHA1), which have also been identified as oncogenes in malignant tumours not only in melanoma cells but also in colorectal or breast cancer cells^32,33^. Because reprogramming of energy metabolism is linked to cell proliferation in cancer^33^, SPANX-A/D proteins, through their interaction with several metabolic proteins, could be essential in the reprogramming of meeting energy demands for cell proliferation and prompt skin tumourigenesis. Overall, SPANX-A/D proteins are emerging as strong candidates for cancer immunotherapy^9,11,12^. In this regard, a more detailed understanding of the pathological role of this protein family in skin tumourigenesis is required to fulfil its therapeutic potential.

Our results provide new molecular insight into the pathological role of SPANX-A/D in carcinogenesis. This protein family plays a multifunctional role in human melanoma, promoting pro-tumoural processes by regulating several hallmarks of cancer, such as nuclear functions and organisation, energy metabolism and cell survival. These findings indicate that the SPANX-A/D protein family may have implications beyond immunotherapy and represent previously unrecognised functions for tumour cell biology, which will be crucial to develop new therapeutic targets for skin tumours.

## Methods

### Cells and culture conditions

In this study, the A375 (CRL-1619; ATCC) and MelHO (ACC-62; Innoprot) human cell lines were used as primary melanoma cell lines, while Colo-800, derived from a human subcutaneous metastatic site, was used as a metastatic cell line (ACC-193; Innoprot). The A375 cell line was cultured in Dulbecco's modified Eagle's medium (Invitrogen, Carlsbad, CA, USA) supplemented with 10% foetal bovine serum (FBS) (GE Healthcare; Chicago, IL, USA), 2 mM l-glutamine (Sigma-Aldrich; San Louis, MO, USA), 100 U/mL of penicillin and 100 μg/mL of streptomycin (Thermo Fisher Scientific (Walthan, MA, USA)). MelHO and Colo-800 cells were cultured in Roswell Park Memorial Institute 1640 Glutamax medium supplemented with 10% FBS, 100 U/mL of penicillin and 100 μg/mL of streptomycin (all from Thermo Fisher Scientific). To study the expression of the SPANX-A/D protein subfamily in other human tumours, HCT-8 (CCL-244; ATCC) and SW480 (CCL-228; ATCC) human colorectal adenocarcinoma cells, MCF-7 (HTB22; ATCC) human mammary adenocarcinoma cells, A2780 (Sigma-Aldrich) human ovary adenocarcinoma cells, HeLa (CCL2; ATCC) human epithelioid cervix carcinoma cells and SMS-KCNR (Texas Tech University) human neuroblastoma cells were analysed. Moderatedly pigmented human epidermal melanocytes (HEMn-MPs) (C1025; Invitrogen) were used as negative control samples, cultured in Medium 254 (Thermo Fisher Scientific) supplemented with 1% human melanocyte growth serum (Thermo Fisher Scientific). Each cell line was cultured according to the manufacturer’s instructions at 37 °C and 5% CO_2_ in a humidified atmosphere.

### Plasmids and transfection

The plasmid encoding YFP–SPANX-A has been previously described^7^. For transfection experiments, A375, MelHO and Colo-800 melanoma cells were seeded onto 12-well or six-well tissue culture plates. For immunohistochemistry experiments, sterile glass coverslips were placed in the wells before cell seeding. Twenty-four hours later, Lipofectamine 2000 reagent (Thermo Fisher Scientific) was used for cell transfection according to the manufacturer’s protocol. To assess transfection efficiency, cells were fixed with 3.7% formaldehyde in PBS for 30 min, and then the coverslips were mounted onto slides using Vectashield Antifade Mounting Medium with DAPI (Vector Laboratories, USA). Image analysis with ImageJ (National Institutes of Health; Bethesda, MD, USA) (1.48v) (https://imagej.nih.gov/ij/) software was used to analyse the intensity of YFP fluorescence.

### Generation of stable SPANX-A/D knockdown (KD) cells

A stable SPANX-A/D knockdown variant of the A375 human melanoma cell line (A375^SPANX-KD^) and a control cell line (A375^CTRL^) were generated using lentiviral infection. Three different SPANX-A/D shRNA- or scramble shRNA (control)-containing lentiviral particles were purchased from OriGene (Rockville, MD, USA). In addition to the shRNAs, these lentiviral particles carry a puromycin resistance gene and the GFP gene, allowing for subsequent selection of infected cells. Infections were performed using polybrene (4 ng/mL) to facilitate the introduction of lentiviral particles into the cells. Cells expressing medium–high levels of GFP fluorescence were selected through cell sorting using a BD FACSJazz (2B/4YG) cell sorter (BD Bioscience, San Jose, CA, USA) and maintained with 5 μg/mL of puromycin (Sigma-Aldrich). To check the efficiency of SPANX-A/D knockdown in A375 cells, Western blot analyses were performed in cells transfected with three different SPANX-A/D shRNAs. The stable cell line was developed using the most efficient SPANX-A/D shRNA. Sustained SPANX-A/D knockdown was periodically confirmed by Western blotting. Three different SPANX-A/D shRNAs (shRNA 1: 5′-CCAAATGGAGGAGGAGGAATTCAT-3′; shRNA 2: 5′-AAGAACATCTCCAGAGGAACTG-3′; shRNA 3: 5′-CTAGTGGTTCGCTACAGGAGGAAC-3′) were used.

### RT-qPCR assays

Total RNA was extracted using a PureLink RNA Mini kit (Life Technologies Inc., CA, USA) according to the manufacturer’s instructions. RNAse-free DNase I was used to prevent DNA contamination. The RNA concentration and purity were assessed by absorbance at 260 nm using a Synergy HT spectrophotometer (Bio-Tek, Winooski, VT, USA). Reverse transcription (RT) was performed in a 20-µL reaction volume with 1 µg of total RNA using an iScript cDNA Synthesis Kit (Bio-Rad, Hercules, CA, USA) to synthesise first-strand cDNA, following the manufacturer′s guidelines. Afterwards, RT-qPCR was performed to check the relative expression level of SPANX-A/C/D genes using tubulin as an internal control. Gene-specific amplification was performed using the CFX96 Real-Time System (Bio-Rad). Quantification was performed using cDNA samples from three separate RNA isolations. The reactions were performed in a total volume of 10 µL containing 50 ng of cDNA, 5 µL of SYBR Green master mix (BioRad) and 200 nM of each primer. The following primers were used: SPANX-A/C/D forward: 5′-AACGAGATGATGCCGGAGAC-3′; SPANX- A/C/D reverse: 5′-TTTGGAGGGGGTTGATTCTG-3′; βIII-Tubulin forward: 5′-CCAGCTGCAAGTCCGAGT-3′; βIII-Tubulin reverse: 5′-CGCCCAGTATGAGGGAGAT-3′.

### Protein extraction and Western blotting

For the protein expression analyses, cells were lysed using ice-cold RIPA buffer, and the protein concentration was determined as previously explained^7^. Protein extracts were diluted in Laemmli sample buffer containing dithiothreitol (DTT) and boiled at 96 °C for 5 min. Thirty micrograms of total protein from melanoma cell lines was loaded onto a 12% resolving gel and transferred to polyvinylidene fluoride membranes. The membranes were blocked with Blotto buffer^7^ for 1 h and then incubated with a rabbit polyclonal anti-SPANX-Antibody (ab119280; Abcam UK; diluted 1:500 in blocking solution), a rabbit polyclonal anti-Lamin A/C (A0249; Abclonal MA USA; diluted 1:1000), a rabbit polyclonal anti-HSPA8 (A14001; Abclonal; 1:500) or a mouse monoclonal anti-alpha tubulin (T5168; Sigma; diluted 1:4000). After washing in Blotto buffer, the membrane was incubated for 1 h with peroxidase-conjugated secondary antibodies (goat anti-rabbit IgG HRP (sc-2004) and donkey anti-mouse IgG HRP (sc-2314), Santa Cruz Biotechnology, TX, USA; diluted 1:1000). Finally, the membranes were evaluated for peroxidase activity by enhanced chemiluminescence with a self-prepared reagent.

### Indirect immunofluorescence and confocal microscopy

Cells growing on sterile glass coverslips were fixed in 4% paraformaldehyde (PFA) for 10 min, permeabilised in 0.2% Triton X-100 for 10 min and blocked for 30 min with 10% (v/v) FBS in PBS. For indirect immunofluorescence analysis, cells were incubated overnight at 4 °C with the following primary antibodies diluted in blocking solution, as indicated: rabbit polyclonal anti-SPANX (ab119280; Abcam, 1:500), rabbit polyclonal anti-Lamin A/C (A0249; Abclonal; 1:50), rabbit polyclonal anti-HSPA8 (A14001; Abclonal; 1:50) and rabbit polyclonal anti-RPL38 (A12038; Abclonal; 1:50). After washing with PBS, the cells were incubated for 1 h at room temperature with Alexa Fluor 488 donkey anti-rabbit IgG (Thermo Scientific; 1:2000) and Alexa Fluor 663 goat anti-rabbit IgG (Thermo Scientific; 1:1000). Specificity controls were performed by omitting the primary antibody before adding the secondary antiserum. Nuclei were stained with Hoechst 33,258 at 10 µg/mL, and the slides were mounted with Fluoromount G (Molecular Probes, OR, USA) as previously described^7^. Samples were analysed using a confocal microscope (Zeiss Apotome 2, Jena, Germany) at the High Resolution Microscopy Core Facility (SGIKER UPV/EHU), and the images were evaluated using ImageJ software (1.48v).

### In gel digestion

To evaluate the expression of the different SPANX isoforms in the A375 melanoma cell line, we followed the protocol described by Urizar-Arenaza et al^7^. Soluble and insoluble protein fractions were loaded onto a precast gradient gel and subjected to reduction, alkylation and digestion using trypsin. Tryptic peptides were extracted from the gel, dried, concentrated and desalted to be separated by liquid chromatography and analysed by tandem mass spectrometry (LC–MS/MS)^7^.

### Analysis of SPANX isoforms by LC–MS/MS

Mass spectrometry analyses were performed as previously explained^7^. Specifically, we used a Q-Exactive HF mass spectrometer (Thermo Scientific, Bremen, Germany) connected to an EASY-nanoLC 1000 System (Thermo) using a nanoelectrospray ion source (Proxeon Biosystems, Odense, DK). Survey full-scan MS spectra (m/z range, 200–2000; resolution 60,000 at m/z 400) were acquired in the Orbitrap followed by the fragmentation of the twelve most intense multiply charged ions. Ions selected for MS/MS were placed on a dynamic exclusion list for 45 s. To improve mass accuracy, internal real-time lock mass calibration was enabled. Additional mass spectrometric parameters included a spray voltage of 2.3 kV, no sheath and auxiliary gas flow, and a heated capillary temperature of 275 °C^7^.

### Cell viability assay

To compare the proliferation of A375^SPANX-KD^ and A375^CTRL^ cell lines, 5 × 10^3^ cells were seeded into 96-well plates in complete medium (DMEM supplemented with l-glutamine and 10% FBS) (Sigma-Aldrich) and incubated overnight. After 18 h, the cells were considered to be in T = 0. After further incubation for 24 h, cell viability was measured using PrestoBlue Viability Reagent (Life Technologies, Thermo Scientific, MA, USA) following the manufacturer’s instructions. Briefly, the medium was replaced with PrestoBlue diluted 1/10 in complete medium and the viability was measured after 2 h of incubation. The results shown are the means of three different experiments, and the error bars indicate the SEM.

### Wound healing assay

The involvement of SPANX-A/D in the motility potential of A375 melanoma cells was investigated using the wound healing assay. A375^SPANX-KD^ or A375^CTRL^ cells were plated in 24-well plates and incubated for 18 h. Next, the medium was replaced with fresh complete medium supplemented with mytomicin (5 µg/mL) (Sigma-Aldrich) to inhibit cell proliferation and incubated for 90 min. A wound was made in each well by scratching with a 200-µl tip, and the medium was again changed to fresh complete medium after extensive washing of the detached cells. Using an inverted light microscope (Zeiss Axioscope), photographs were taken at T = 0 and after 24 h of incubation (T = 24 h), and the wound area was measured using ImageJ software (1.48v) to establish the initial wound area. The closed wound area was calculated as the wound area at T = 0 minus the area at T = 24. The results shown are the means of three independent replicates, and the error bars indicate the SEM.

### Transwell migration assay

The role of SPANX-A/D proteins in tumour cell migration was examined using Transwell migration assays. In total, 1.5 × 10^4^ A375^SPANX-KD^ or A375^CTRL^ cells were seeded on top of 8-µm pore membrane inserts (Falcon) in complete medium. The cells were allowed to migrate overnight for 20 h. Next, the inserts were fixed in 4% PFA, rehydrated in PBS and stained using 0.5% crystal violet (Sigma-Aldrich) in PBS for 15 min. For the quantification of cell migration, each insert was examined under a light microscope, and the number of cells in six randomly chosen fields was counted. The results are expressed as the average number of migrated cells in a 20 × field. The results shown are the means of two different experiments, and the bar errors indicate the SEM.

### Co-immunoprecipitation and in-solution digestion

For the SPANX-A interactome, A375 melanoma cells were transfected with the YFP-SPANX-A plasmid as described above. After protein extraction using Co-IP buffer (100 mM NaCl, 20 mM Tris–HCl, 1% NP-40, and complete protease inhibitor cocktail (Complete tablets, Roche)), the pull down was performed in quadruplicate with GFP-Trap_Magnetic Agarose (Chromotek, Germany) following the manufacturer’s instructions. The precipitated protein complexes were independently recovered, washed and eluted with guanidinium hydrochloride 8 M pH 8 at 70 °C for 15 min. The eluted proteins were then reduced, alkylated and subjected to in-solution digestion with trypsin (Roche Diagnostics) overnight at 37 °C. The resulting peptides were desalted using C18 Micro SpinColumns (Harvard apparatus), dried in a speed-vac centrifuge (Thermo Scientific) and resuspended in 0.1% FA before LC–MS/MS analysis.

### Interactome analyses by LC–MS/MS

Mass spectrometry analyses were conducted using a Q-Exactive HF-X mass spectrometer (Thermo Scientific, Bremen, Germany) connected to an EASY-nanoLC 1200 System (Thermo) using a nanospray flex ion source (Thermo). Desalted peptides were loaded onto an Acclaim PepMap100 precolumn (75 μm × 2 cm, Thermo Scientific) connected to an Acclaim PepMap RSLC (75 μm × 25 cm, Thermo Scientific) analytical column^34^. To elute peptides from the column, we used the following gradient: 120 min from 2.4 to 24%, 2 min from 24 to 32% and 12 min at 80% acetonitrile in 0.1% formic acid at a flow rate of 300 nL min^−1^. Full MS scans were obtained from m/z 375 to 1800 with a resolution of 60,000 at m/z 200. The 10 most intense ions were fragmented by higher energy *C*-trap dissociation with a normalised collision energy of 28, and MS/MS spectra were documented with a resolution of 15,000 at m/z 200. The maximum ion injection times were 50 ms and 100 ms, whereas AGC target values were 3 × 10^6^ and 1 × 10^5^ for survey and MS/MS scans, respectively. To avoid repeat sequencing of peptides, dynamic exclusion was applied for 20 s. Singly charged ions, ions with unassigned charge states and ions with charge states above 5 were also ignored from MS/MS. Data were obtained using Xcalibur software (v.4.0) (Thermo Scientific)^34^.

### Data processing and bioinformatics

For the SPANX-A/D isoform analyses, raw files were searched against the combined human database 2015.08 UniProt (with 42,122 sequence entries) and TrEMBL (with 49,496 sequence entries) using the MaxQuant platform version 1.5.2.8 with an Andromeda search engine. To study the SPANX-A/D interactome, raw files were searched against the UniProt-SwissProt human database (version 2018_11, 42424 entries) using the MaxQuant^35^ platform (version 1.6.0.16) with its internal search engine Andromeda. The precursor and fragment tolerances were 4.5 and 20 ppm, respectively. A peak list was generated using the Quant element of MaxQuant and the following parameters: a maximum of 2 missed cleavages were allowed, and enzyme specificity was set to trypsin. Additionally, carbamidomethyl (C) was chosen as a fixed modification and variable modifications included oxidation (M), deamidation (NQ) and phospho_STY (STY). The peptide and protein FDR were 0.01, the site FDR was 0.01, the max. peptide PEP was 1, the min. peptide length was 7, and the min. unique peptides and peptides were 1. For protein quantitation in interactome studies, only unmodified peptides were used. The match between runs option was enabled with a 1.5-min match time window and a 20-min alignment window to match identification across samples, and normalised spectral protein label-free quantification (LFQ) intensities were calculated using the MaxLFQ algorithm. According to the protein group assignment performed by MaxQuant, the identified proteins were determined after removing the contaminants, reverse hits and proteins identified only by site. We only considered proteins identified with ≥ 2 peptides and ≥ 1 unique peptide. Additionally, phosphopeptide data were filtered by FDR < 1%, and only the phosphosites displaying a localisation probability above 0.75 were considered confident phosphorylated sites (Class I sites). From all detected proteins, we considered putative SPANX-A interactors that were identified in at least three of the four YFP-SPANX-A pull-downs. The PANTHER (v13.1) functional annotation tool (http://geneontology.org/) was used to detect the overrepresented gene ontology (GO) terms “biological process” and “molecular function” within the possible SPANX-A interactors.

## Supplementary Information

Supplementary Information 1.

Supplementary Information 2.

Supplementary Information 3.
