# Supplementary material for: The multifunctional role of SPANX-A/D protein subfamily in the promotion of pro-tumoural processes in human melanoma
Source: Sci Rep. 2021 Feb 11;11:3583. doi: 10.1038/s41598-021-83169-1 (PMC7878863; doi:10.1038/s41598-021-83169-1)
Supplement: Supplementary file 1 — Supplementary Information 1. [file 41598_2021_83169_MOESM1_ESM.pdf]

The multifunctional role of SPANX-A/D protein subfamily in the promotion of pro-tumoural processes in human melanoma

Itziar Urizar-Arenaza1,2,#, Aitor Benedicto[3], Arantza Perez-Valle[3], Nerea Osinalde[4], Vyacheslav Akimov[5], Iraia Muñoa-Hoyos[1,2], Jose Antonio Rodriguez[6], Aintzane Asumendi[3], Maria Dolores Boyano[3], Blagoy Blagoev[5], Irina Kratchmarova[5] and Nerea Subiran[1,2,#].

1 Department of Physiology. University of the Basque Country (UPV/EHU), Leioa, Spain
2 Biocruces Bizkaia Health Research Institute. Bizkaia. Spain
3 Department of Cell Biology and Histology. University of the Basque Country (UPV/EHU), Leioa, Spain
4 Department of Biochemistry and Molecular Biology. University of the Basque Country (UPV/EHU), Vitoria-Gasteiz, Spain
5 Department of Biochemistry and Molecular Biology. University of Southern Denmark. Odense. Denmark
6 Department of Genetics, Physical Anthropology and Animal Physiology, University of the Basque Country (UPV/EHU), Leioa, Spain

# To whom correspondence should be addressed:

• Nerea Subiran Ciudad, Department of Physiology. University of the Basque Country. 48940. Leioa, Bizkaia, Spain. +34 946015673. nerea.subiran@ehu.eus

•Itziar Urizar-Arenaza. Department of Physiology. University of the Basque Country. 48940. Leioa, Bizkaia, Spain. +34 946012844. Itziar.urizara@ehu.eus

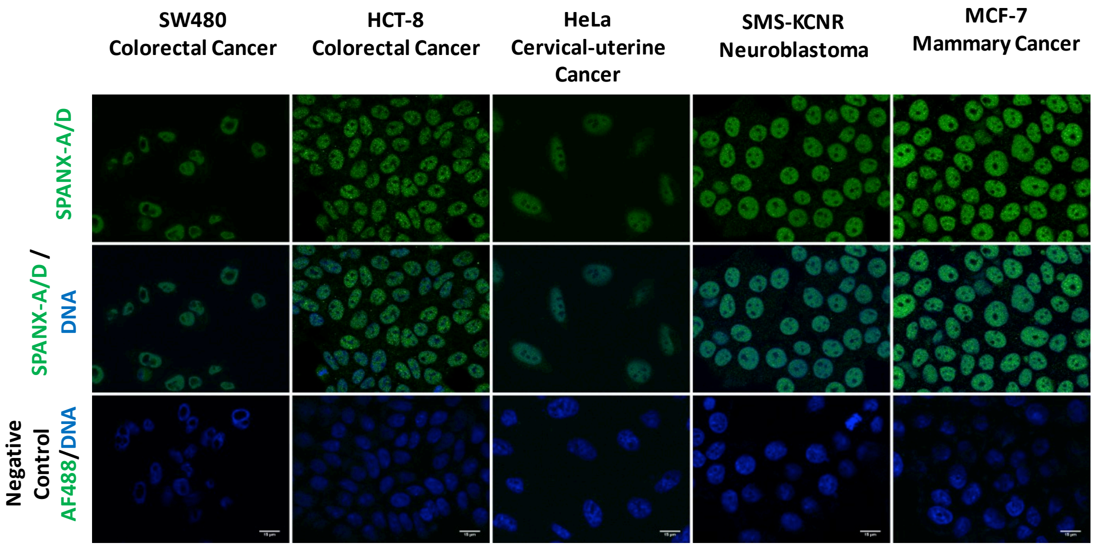

Supplementary Figure 1. Characterisation of SPANX-A/D protein family in cancer cells by inmunocytochemistry. Human colorectal adenocarcinoma cells (SW480 and HCT-8), human epitheloid carcinoma cells (HeLa), neuroblastoma cells (SMS-KCNR), human mammary adenocarcinoma cells (MCF-7) and human ovary adenocarcinoma cells (A2780) were stained with the anti-SPANX antibody and the staining was analyzed by confocal microscopy. For the specificity of the secondary antiserum, the primary antibody was omitted. The nuclei were stained with Hoechst and are represented in blue. Scale bar 15 µm. (N=3). AF488: Alexa Fluor 488 donkey anti-rabbit IgG (Thermo Scientific).

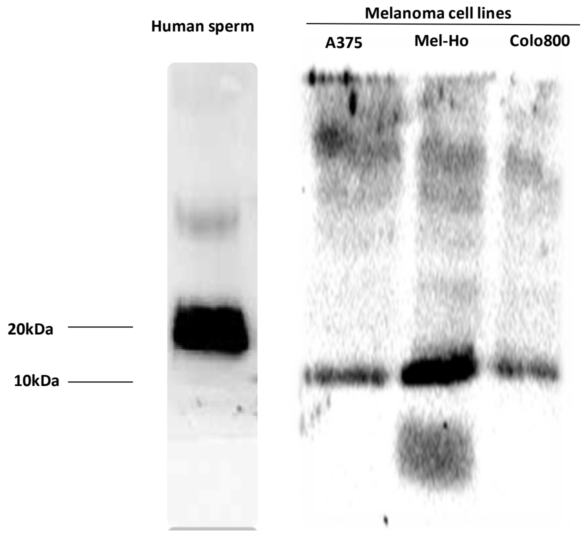

Supplementary Figure 2. Characterisation of SPANX-A/D protein family in human melanoma cancer cells by Western Blot. Human sperm lysate was used as positive control (left panel). A375, MelHo and Colo800 human melanoma cells were used (right panel). N=3.

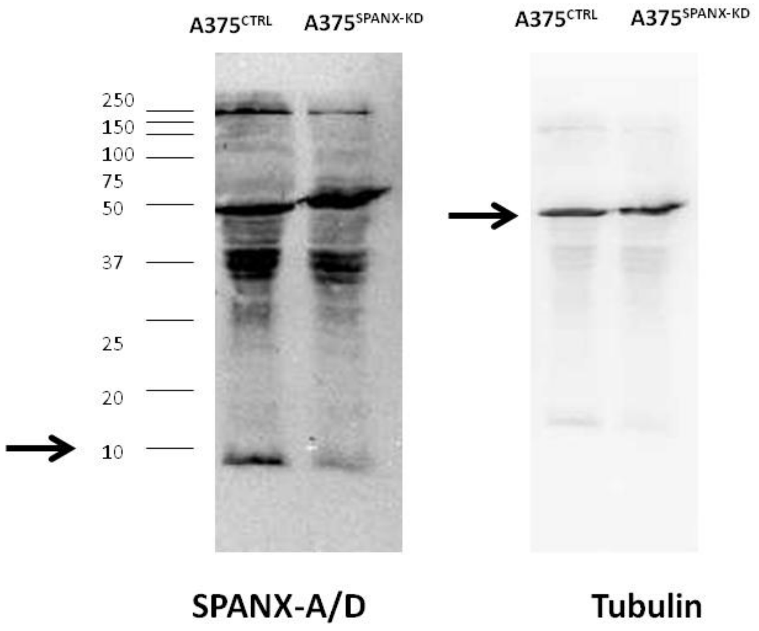

Supplementary Figure 3. Silencing of SPANX-A/D at protein level by Western Blot. N=3.

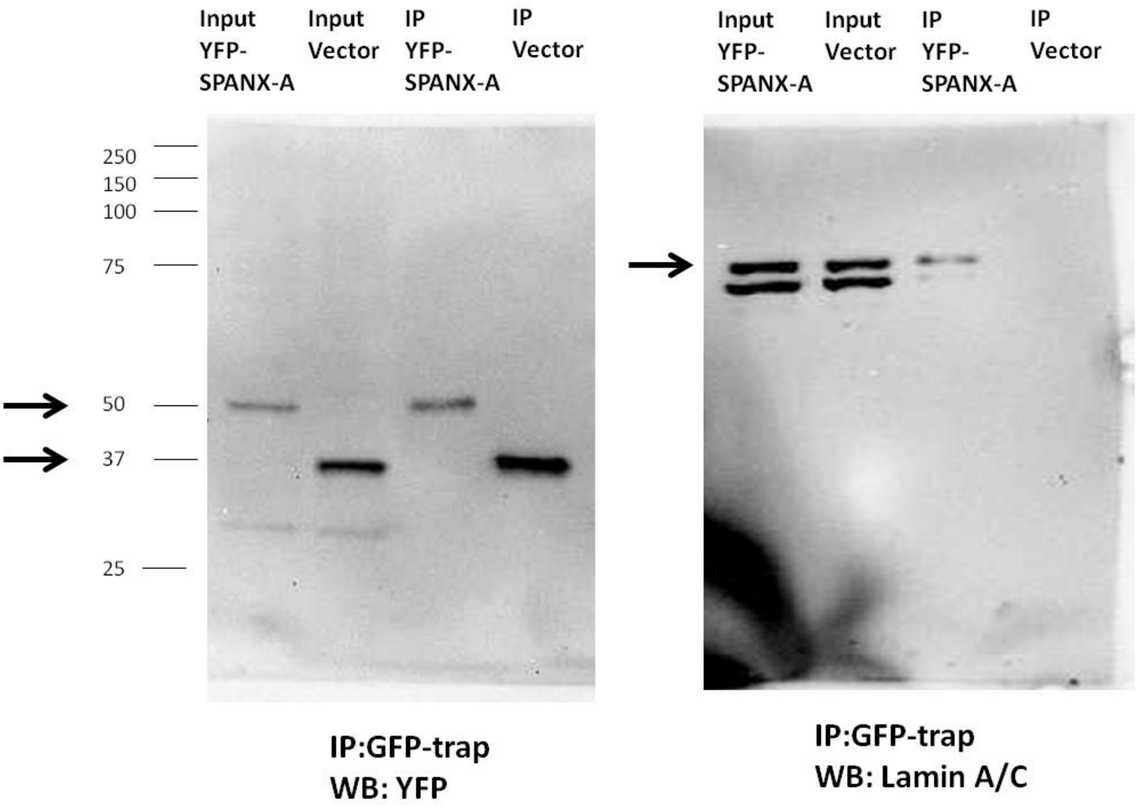

Supplementary Figure 4. Study of the interaction between YFP-SPANX-A and Lamin A/C in A375 melanoma cell line by Western Blot. Immunoprecipitation of YFP-SPANX-A in A375 melanoma cell line (left panel). Co-immunoprecipitation of YFP-SPANX-A and Lamin A/C. As negative controls A375 cells were transfected with the YFP empty vector. N=3.

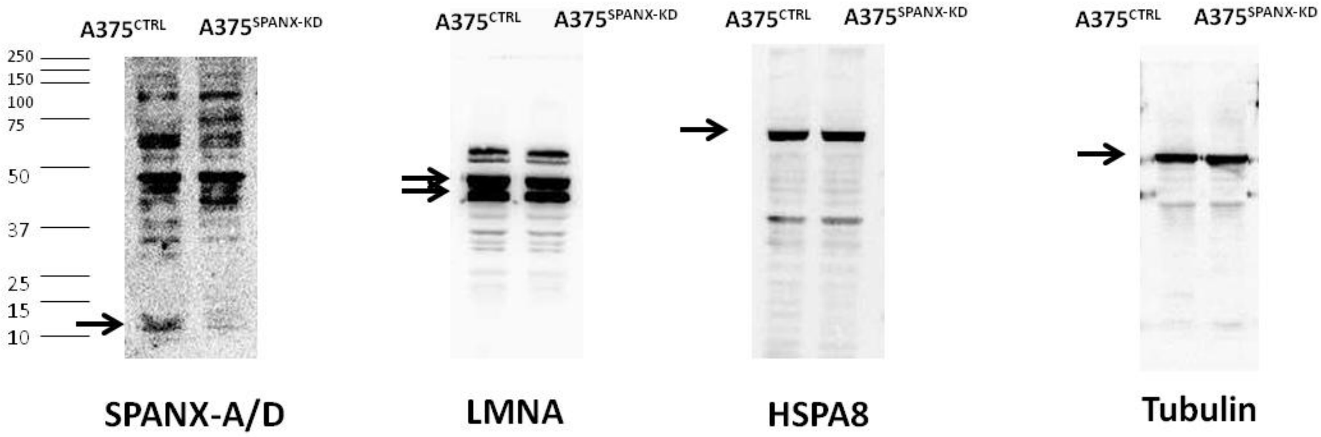

Supplementary Figure 5. Expression of Lamin A/C and HSPA8 in A375$^{SPANX\text{-}KD}$ cells. A375$^{SPANX\text{-}KD}$: Stable SPANX-A/D knockdown variant of the A375 human melanoma cell line; A375^{CTRL}: control cell line. N=3.
